# Material Behavior of PIR Rigid Foam in Sandwich Panels: Studies beyond Construction Industry Standard

**DOI:** 10.3390/ma17020418

**Published:** 2024-01-14

**Authors:** Sonja Steineck, Jörg Lange

**Affiliations:** Institute for Steel Construction and Materials Mechanics, Technical University of Darmstadt, 64287 Darmstadt, Germany

**Keywords:** sandwich panels, PIR-rigid foam, orthotropic material, Hill plasticity, Tsai–Wu failure criterion

## Abstract

A deep understanding of the material parameters and the behavior of sandwich panels, which are used in the construction industry as roof and façade cladding, is important for the design of these construction components. Due to the constant changes in the polyurethane (PU) foams used as a core material, the experimental database for the current foams is small. Nowadays, there is an increasing number of failures of façade and roof panels after installation. This article presents a variety of experimental investigations on sandwich panels from two manufacturers with a core of polyisocyanurate (PIR) rigid foam (density: 40 kg/m^3^). As part of this study, compression, tension, shear, and bending tests were performed in several spatial directions and over the range required by the standard. The results of the tests showed the orthotropy of the core material and the dependence of the material on the direction and type of load. The stress-strain curves showed linear and non-linear areas. Using the data from this experimental study, a numerical model was implemented which utilized the Hill yield criterion to represent the orthotropy of the core material. The present investigation suggests that the classical von Mises failure criterion, used in many studies, is not suitable for the foam system applied in these sandwich panels. Instead, the Tsai–Wu criterion is more appropriate for defining the failure stresses.

## 1. Introduction

**Figure 1:**
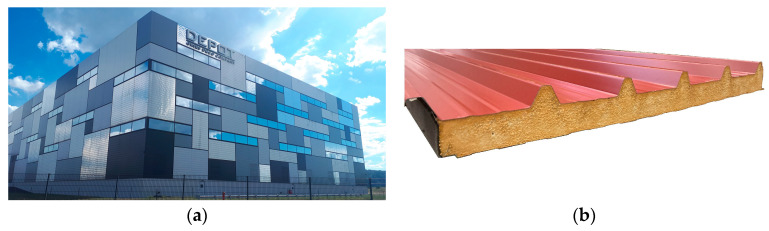
Sandwich panels in the construction industry: (**a**) Industry building with sandwich panels, and (**b**) Roof sandwich panel with PU rigid foam.

In the construction industry, sandwich panels are mainly used as roof and façade components (see Figure 1a). Due to the good strength-to-weight ratio and the short construction time, these panels are particularly popular and widely used in industrial and hall constructions. In this area of application, sandwich panels consist of two thin steel faces with a thicker core in between. The core material can be a polyurethane (PU) rigid foam, with a density of around 40 kg/m^3^, or mineral wool. Mineral wool is mainly used for high fire protection and acoustic insulation requirements. In most cases, PU rigid foams are used (see Figure 1b) [1]. The PU rigid foam type currently used in sandwich panels is primarily polyisocyanurate (PIR) rigid foam. It is a closed-cell foam with good physical (e.g., thermal insulation) and mechanical properties (strength and stiffness) compared to other façade components. In the following, the initials PU include both the current PIR foam system and the foams known in the past as PUR (polyurethane).

The first industrial production of sandwich panels for construction took place in the 1960s [2]. Since then, many studies have been carried out on laminate structures in general and some research has been carried out on sandwich structures as used in construction. In the basic literature [3,4,5,6,7,8], sandwich theories for calculation are presented. Here, it is assumed that the PU core behaves in a linear-elastic, homogeneous, and isotropic way. The loads are distributed to the steel faces and core material by normal and shear stresses. The face sheets take on the bending stresses and are therefore loaded with normal stresses. The core material is stressed by shear force and, in simplified terms, receives a constant shear stress distribution over the core height.

In addition to this basic literature, which is used for the design, there is a significantly smaller amount of research work that deals with a more detailed consideration of the stress-strain relationships in sandwich panels with PU-rigid foam for the construction industry.

Menges and Knipschild [9] established mathematical approaches for the correlation between the isotropic cellular structure and the mechanical properties of the foam material. Gibson, Ashby, and Medalist [10,11,12] established a relationship between the density of cellular foams and mechanical properties, such as stiffness and strength. A detailed knowledge of the solid content and the cell structure in the foam system is necessary to apply the established relationships.

Thermann [13] and Zhao [14] contributed to the calculation of the wrinkling stress of inhomogeneous or anisotropic sandwich panels. During wrinkling, the compressed face sheet, which is supported by the core, fails in terms of stability. As a result, the face sheet wrinkles. In their publication in 2012, Hassinen and Misiek [15] show the inhomogeneity and anisotropy of a polyurethane core material and provide a simplified calculation of the wrinkling load-bearing capacity for an orthotropic core material. The wrinkling failure of the compressed face sheet is still declared as the usually decisive and design-relevant failure. However, the calculation in the responsible standard, EN 14509 [16], for this failure is based on the assumption of a homogeneous and isotropic core layer.

Furthermore, several approaches to laminate theories and higher-order sandwich theories have been developed [17,18,19], which can also be applied to the sandwich panels investigated here. However, the application is demanding and again requires a precise knowledge of the anisotropy and inhomogeneities prevailing in the core material.

Numerical investigations have been carried out in parallel in several areas. The most recent investigations are, for example, by Pozorski and Porzorska [20,21,22]. In their publications, they described different ways of implementing the PU rigid foam core. In addition to the two classic elastic-plastic isotropic and orthotropic material models, they also investigated a model that uses the Hill yield criterion for the hardening range. Furthermore, they performed calculations with models for hyper-elastic and crushable foams [20]. In [21], 3600 open regular hexagonal shapes, representing the cellular structure, were implemented for a numerical investigation. The cell walls were neglected due to their minor influence. The 2D model was examined with regard to compressive, tensile, and shear loads. During the modeling process, various challenges arose in the selection of suitable geometry and material parameters, such as Young’s modulus and Poisson’s ratio. The authors also showed that a calculation based purely on compressive stresses is inadequate for indentations. Instead, the interaction of normal and shear stress should be taken into account in order to map the real stress distribution in these areas [22].

Experimental investigations on sandwich panels with steel facings and a PUR core were presented by Baehre in a research report in 1989 [23]. The focus of this work was on the wrinkling failure mode of the compressed face sheets.

Gdoutos und Daniel [24,25,26] published test results on composite sandwich beams with unidirectional carbon/epoxy facings and divinycell foams (PVC) (100 kg/m^3^ and 250 kg/m^3^). They described the influence of material and geometry non-linearity on the load-deformation behavior of sandwich panels. Tests were carried out where the deformation of the core was monitored with birefringent coatings using reflection photoelasticity. They show that the shear stress distribution in the core deviates from its constant course as the load increases and reaches a maximum in the middle of the height of the beam. The start of the deviation from the proportional stress-strain course coincides with the start of the non-uniform stress distribution over the thickness. They observe a stronger deviation from a linear-elastic load-deformation curve for short beams with high shear loads. In the case of multiaxial loading of the core material, they describe the stress failure using the Tsai–Wu failure criterion. This failure criterion can be used for anisotropic materials. They differentiate between the failure modes of wrinkling of the face sheets and the shear failure of the core layer.

Sokolinsky et al. [27] studied sandwich beams with aluminum facings and a PVC foam core. They compared the experimental results with the approaches of the classical sandwich theory and with a high-order sandwich panel theory. Linul and Marşavina [28] carried out investigations on sandwich panels with polymeric core materials (40 kg/m^3^ and 200 kg/m^3^) and face sheets of glass-fiber reinforced polymer (GFRP), polyester, epoxy, and aluminum. They established failure-mode maps based on their analytical and experimental investigations. Vălean et al. [29] considered sandwich panels with a rigid PUR foam core (40 kg/m^3^) and steel facings. They carried out bending, tensile, and compression tests in accordance with EN 14509: 2013 [16] and characterized the material in the thickness direction.

Due to the high variability of the material properties, the standard for sandwich panels in the construction standard, EN 14509, provides the basis for test-based approvals. Based on the data from the tests, approvals are generated using the 5% fractile values, which contain minimum values for the material parameters. These approvals must be renewed on the basis of tests if the geometry or foam composition changes.

The literature listed here shows that there is a large number of analytical approaches for the more accurate design of sandwich panels. However, the focus of most research work is on the wrinkling failure mode. Failure of the core material is usually only dealt with to a minor extent. The experimental database for the sandwich panels used in the construction industry is also rather small. While analytical approaches from other sectors (such as the automotive and aircraft industries) can be transferred to sandwich structures used as façade and wall panels in the construction industry, it is not possible to transfer material-specific material parameters from experimental studies. Furthermore, due to the constant and ongoing development of the core material, in particular the development from PUR to PIR in the last 10 years, previous experimental data sets cannot be directly transferred to the currently produced sandwich panels [30].

This article deals with this problem and examines current sandwich panels with a PIR rigid foam core that are commonly used in the construction industry. A large experimental study is carried out to determine the material characteristics beyond the scope described in the standard EN 14509 report. The focus is on the consideration of the orthotropy of the core material and its behavior under different loads. The individual relationships between load direction, production direction, and cell geometry are pointed out. This is followed by a numerical investigation based on the test results, which illustrates the difficulties and possibilities of implementing the core material in an FE model. In the following discussion, the focus is placed on core failure in addition to the already much-investigated wrinkling failure. In contrast to some other research work, the Tsai–Wu hypothesis is used to determine the stress failure in the core. This takes into account a possible increase in stress absorption under additional shear stresses.

## 2. Materials and Methods

### 2.1. Materials

The tests published in this paper were carried out on sandwich panels manufactured for use as roof and façade components in the construction industry. The properties of the core material vary depending on the production process (foam mixture, temperature, and geometry) and the environmental conditions [30,31,32,33]. The production of sandwich panels was a continuous production line on a double-belt system. The outer and inner face sheets were unwound from coils and profiled. The PU foam was injected between the heated face sheets, which moved at a speed of 6 to 8 m per minute. The exothermic reaction of polyol with isocyanates, and various additives, during the production process resulted in the formation of cells in which blowing agents in the form of pentane were trapped. The PU rigid foam was therefore a closed-cell material [2,34,35].

The tests presented here include two manufacturers: Manufacturer A and Manufacturer R. Table 1 describes the properties of the sandwich panels tested.

**Figure 2:**
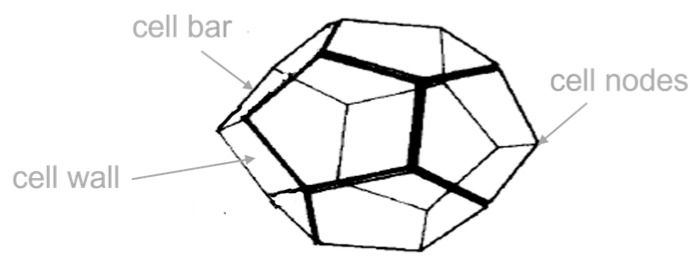
Simplified schematic representation of cell elements in the form of a pentagonal dodecahedron with thick cell bars and thin cell walls, based on [9,34].

Microscope images of the foam show that the largest proportion of the PU solid was located in the cell bars and cell nodes (see Figure 2) [9,15,36]. The very thin cell walls were located between the cell bars and did not contribute to the load transfer. The cell bars are mainly responsible for load transfer [9]. Due to the continuous production, the cells were slightly longer in the longitudinal direction of the panel (production direction) than in the other spatial directions (see Figure 2) [36]. The dimensions and orientation of the cells in the y- and z-direction were similar. In general, the cells were in the form of a pentagonal dodecahedron (see Figure 2).

The focus of this paper is on the description of the core material. The face sheets were removed for some experiments. The thickness of the core material was also partially changed in order to produce square test specimens. The number of tests varied between 3 and 12 samples for each test configuration. In total, more than 200 tests were carried out on the sandwich panels listed in Table 1.

### 2.2. Methods

Various tests based on the EN 14509: 2013 standard [16] were carried out on test specimens to determine the material parameters and material behavior. The small-scale compression and cross-panel tensile tests (cube tests), the shear beam tests, and the bending tests on the entire panel are described in more detail below. To obtain more precise information, the tests were carried out beyond the requirements of the standard. This includes experiments in different test directions as well as testing beyond the limits specified in the standard.

#### 2.2.1. Compression Test

To determine the compression parameters, the tests were carried out in accordance with EN 14509: 2013 Section A.2. The sample dimensions were usually 100 mm × 100 mm × element height.

**Figure 3:**
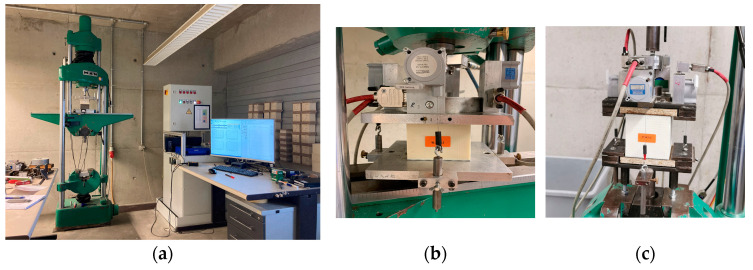
Experimental apparatus and setup: (**a**) MAN 100 kN testing machine for the compression and tensile tests with measuring and control electronic; (**b**) Compression test setup; and (**c**) Tensile test setup.

The tests were carried out using a MAN 100 kN universal testing machine (see Figure 3a). The displacements were recorded with one cable displacement transducer on each side of the test specimen. For the subsequent evaluation, the four displacements were averaged. The load was recorded using a load cell (10 kN, HBM U2B) (see Figure 3b).

The tests were carried out at a speed of 3% of the specimen height per minute and were run up to 10% compression in accordance with the standard. The resulting strength at this time was used as the maximum compressive strength (f_Cc_). The compression modulus of the core material (E_Cc_) was determined via the slope in the linear elastic range. According to the standard, only the compression test in the direction of the element height (z-direction) is intended.

As biaxial stress states also occur in the core material of sandwich panels, and the aim was to describe them as close to reality as possible, compression tests were carried out in all three spatial directions: along the longitudinal axis of the panel (x), over the width of the panel (y), and over the thickness of the panel (z). The face sheets were removed and in deviation from the standard. These compression tests were carried out up to a compression of 80% to 90%, or until failure. The speed was set to 10 mm/min to reduce the duration of the tests. In previous tests, it was found that the increased speed had no significant influence on the results, which are presented here. In some cases, the sample measurements deviated from the mentioned 100 mm × 100 mm, as the aim was to achieve a cube geometry. This was particularly important for sandwich panels with a height deviating from 100 mm.

#### 2.2.2. Tensile Test

To determine the tensile parameters, cross-panel tensile tests were carried out on cubes with dimensions of 100 mm × 100 mm × element height (analogous to EN 14509 Section A.1.). The same testing machine was used as in the compression tests (see Figure 3a). To ensure a uniform load application, plates were glued (with 2K, epoxy L + EPH 161) to the face sheets, and the load was applied on both sides of these plates (see Figure 3c). Each test was carried out at a speed of 3% of the specimen height per minute. The test was run until the specimen failed in tension. The tensile modulus was determined using the gradient in the linear range between 20% and 40% of the maximum load [16].

To carry out tensile tests in all three spatial directions, the face sheets were removed and cubes with a side length of 100 mm were cut from the core of an element. The plates for load application were glued (2K, epoxy L + EPH 161) directly onto the foam.

#### 2.2.3. Shear Test

**Figure 4:**
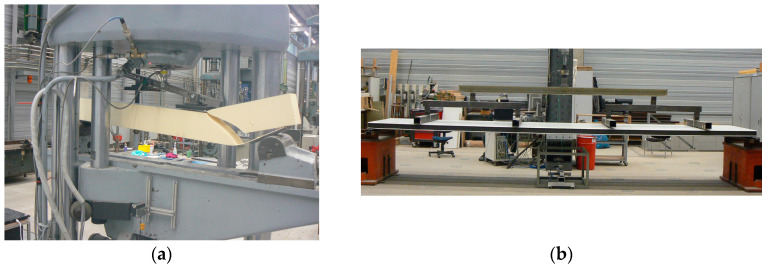
Experimental setups: (**a**) Shear test setup with a typical shear pattern followed by delamination of the lower face sheet; and (**b**) Six-point bending test setup on a whole panel.

The characteristic values for shear were determined in the test on short beams analogous to EN 14509 Section A.3 with a width of 100 mm. The length of the shear beams depended on the thickness of the used panel and varied accordingly in the range from 900 mm to 1400 mm. The two loading points of the four-point bending test were located in the third point of the beam. The tests were carried out using a MAN 1 MN universal testing machine (see Figure 4a). The displacements were recorded with one displacement transducer (IL300) in the middle of the beam on each side of the test specimen. For the subsequent evaluation, the two displacements were averaged. The load was recorded using a load cell (10 kN, HBM U2B).

According to the standard, only tests along the longitudinal axis of the panel are generally required. These show the shear properties in the xz-plane. Tests transverse to the production direction can be carried out with a maximum length of the component width. Profiling must be considered during the evaluation. The properties are obtained in the yz-plane.

#### 2.2.4. Bending Capacity Tests

Figure 4b shows a full-scale panel test according to EN 14509 Section A.5. Entire components with a length of 4 to 8 m were loaded by a servo-hydraulic cylinder in a six-point bending configuration. The deflection in the middle of the beam was measured by two optical displacement transducers (IL 300). Optionally, the face sheet elongation could be recorded with a strain gauge. The load cell (Type K GTM) had a nominal load of 150 kN. These tests were carried out to determine the maximum bending capacity of a single-span beam. Under normal conditions (room temperature and no aging), wrinkling was usually the decisive and desired failure.

## 3. Results

The results described below show extracts from a series of tests on samples from manufacturers A and R with a total of over 200 individual tests. However, as described in the beginning, the results for the material parameters are subject to a large scatter and are highly dependent on the manufacturer and the production process. A direct transfer of the individual results shown by any sandwich panels should therefore only be carried out with caution.

The results of the tests in different spatial directions are of particular importance. There is hardly any current experimental data in this field, and the results presented below represent an important milestone of detailed research into sandwich panels for the construction industry.

### 3.1. Compression Tests

**Figure 5:**
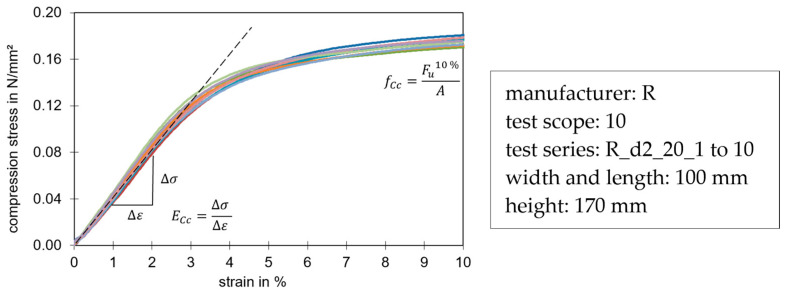
Typical stress-strain diagram of a compression test in z-direction. The different colors represent the individual trials of the test series.

Figure 5 shows a stress-strain diagram of compression tests in the z-direction (panel thickness), up to a compression of 10% in accordance with the standard. In this figure, the determination of the compression stiffness and strength can be observed.

**Figure 6:**
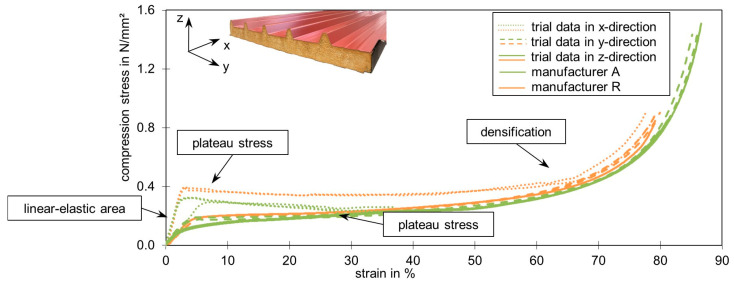
Stress-strain diagram of compression tests in three spatial directions on 13 samples from two different manufacturers (A and R).

Figure 6 shows the stress-strain diagram of all three directions on samples from two manufacturers (A and R), up to a strain of almost 90%. Considering first the tests were loaded in the z- and y-directions, we saw very similar stress-strain curves. There were three sections within each curve. The first was a linear-elastic area up to a compression of about 3%. This was followed by a non-linear decrease in stiffness to a minimum, which led to large deformations (plateau stress). Densification began at around 55% strain and resulted in a rapid increase in stress and stiffness. This behavior is typical for elastomeric foams [12] and can also be described as hyper-elastic. With the unloading curves, the test specimens almost return to their original dimensions.

The curves of the samples loaded in the x-direction differed from the curves of the other load directions. The foam system behaved stiffer in the linear-elastic range and reached a maximum value at around 2% strain. After this, the stress initially dropped to a plateau until a densification process occurred at around 55% strain. Such a stress-strain diagram characterizes elastic-plastic foams [12].

**Figure 7:**
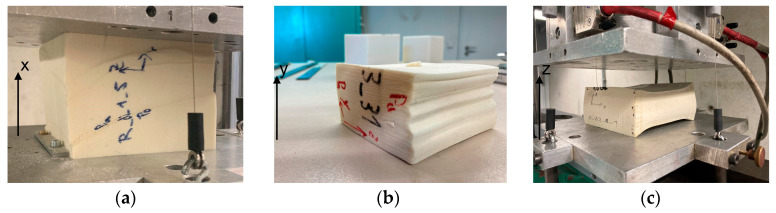
Optical appearances due to high compression: (**a**) In the x-direction, yield lines occur, which lead to fracture of the foam system at higher compression; (**b**) In the y-direction, the test specimen can be compressed by up to 85% without visually recognizable foam fractures. Bulges form on the side treated with primer; (**c**) In the z-direction, a relatively uniform compression occurs.

Although only the stress-strain curves of the tests in the x-direction deviated significantly from the other ones, all test samples showed optical characteristics according to their direction (see Figure 7). In the x-direction, yield zones occurred even at small strains, which became visible in the foam (see Figure 7a). Nevertheless, the foam system reacted stiffer on this axis and foam fractures occurred. In the y-direction, the foam was compressed by up to 90% without visible cracks. If the face sheets were cut off directly underneath, then the influence of the primer on the deformation could be monitored. On the side of the primer, many bulges formed under load in the y-direction (see Figure 7b). In the z-direction, the foam deformed evenly under load and could be compressed by up to 90% without visible foam cracks. A high proportion of elastic deformation could be observed in all directions (Figure 7c). All foam systems returned to about 80% of their original dimensions after unloading and a short rest period.

**Figure 8:**
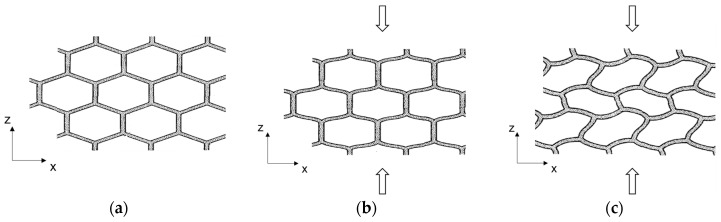
Simplified schematic representation of (**a**) cell geometry and orientation in the unloaded state, (**b**) bending of the cell bars in the linear-elastic range under compression load. The deformation is proportional to the load, and (**c**) non-linear deformation of the cells under compression load. with a large deformation at almost constant load occurs due to buckling of the cell bars and walls. Based on [11].

The strong differences in the behavior of the various directions observed in the tests can be explained by the pronounced orthotropy of the core material (Figure 2 and Figure 8a). During the load increase, the cell bars initially bent in the y- and z-directions, which was characterized by the linear increase in deformation (see Figure 8b). In the predominantly plateauing area, with its characterized low gradient, the cell bars buckled (see Figure 8c). Occasionally, the thin cell walls may tear open. When the bulging gets severe, so that the opposing cell bars abut and touch each other, the densification begins and the stress increases [37,38]. The stress-strain curve then behaves analogously to that of the solid material of the cells [25] and the cell walls rupture completely. However, the blowing agent pentane escaped from the sample when the pressure was released. This was recognized by the smell that emerged from the sample at this point.

Due to the elongated shape of the cells, there was more solid material (cell bars) in the x-direction. As a result, the stiffness due to the bending of the cell bars was significantly greater than in the y- and z-direction. However, when loaded in the x-direction, the cell bars aligned in the direction of force were also significantly longer and were subjected to greater compression. The bending of the cell walls was followed by a stability failure of the cell bars in the x-direction. However, joints formed at the nodes of the cell walls before this. If these plastic hinges formed at several cells along the cross-section, the visually recognizable yield zones could be observed (see Figure 7a). Foam fractures occurred at the edges of the specimen. The densification in this load direction started when the cell bars touched each other. The escape of the blowing agent could already be detected during the tests, as the cells opened due to the bursting of the cell walls when compression was applied in the x-direction and recognizable foam fractures occurred.

### 3.2. Tensile Tests

**Figure 9:**
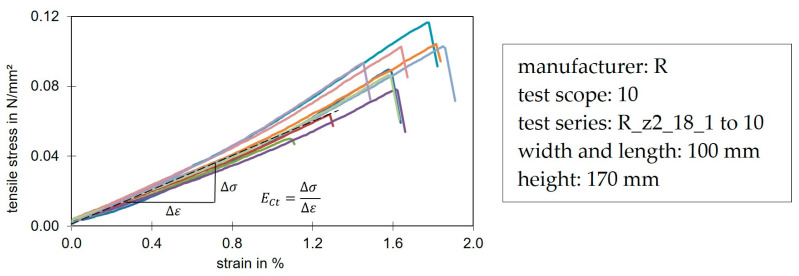
Typical stress-strain curves of an elastic-brittle foam under tension in z-direction. The different colors represent the individual trials of the test series.

Figure 9 shows a typical associated stress-strain distribution from tensile tests along the z-direction according to the standard. The graphs are almost linear-elastic up to failure. The failure was typically a foam fracture near the production top side. Under tension, PIR behaved in a brittle way and thus exhibited a different material behavior than under compression. Also typical for the investigated foam systems was the higher compressive strength at 10% compression compared to the tensile strength at failure.

Compared to the compression tests, the tensile tests generally showed a higher scattering at the maximum strength. This was due to the sensitivity of the tensile tests to blow holes (defects in the foam) and the inhomogeneity of the foam across the width and height of the panel. Compared to the tensile strength, the stiffness did not scatter significantly.

**Figure 10:**
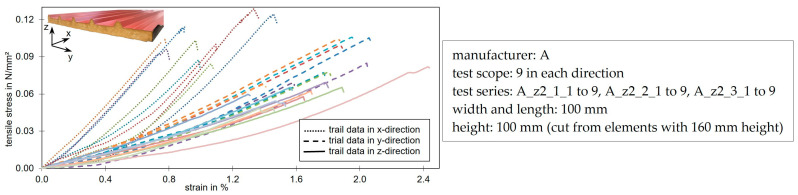
Stress-strain diagram of tensile tests in 3 spatial directions. The different colors represent the individual trials of the test series.

Figure 10 shows the stress-strain curves of tensile tests in the x-, y- and z-direction. The foam behaved in a brittle way under tensile load in all three directions. However, differences could be seen in the mean values of the tensile stiffness and strength as well as in the elongation at failure. These results are consistent with the findings from the compression tests.

The greatest stiffness and strength were obtained in the x-direction. The load was introduced into the long and numerous cell bars which were predominantly aligned parallel to this direction. The elongation was low because the long cell bars were mainly loaded in tension and the neighboring short cell bars only experienced little bending before they finally failed. The bending of the cell bars near the cell nodes was mainly responsible for the linear deformation. Analogous to the results from the compression tests, the results in the y- and z-directions were more similar than those in the x-direction. They showed lower stiffnesses and tensile strengths. In the tests shown here, it is noticeable that the stiffness in the thickness direction (z-direction) was on average 24.5% lower than in the transverse y-direction. The strength was also on average 28.7% below the tensile strength in the y-direction. The higher elongation compared to the x-direction was due to the stronger branching of the cells along the two other load directions. This results in increased bending of the cell bars.

Similar to the compression tests, the tests with the load along the x-axis achieved the highest strength values, followed by the ones with the load in the y-direction. The lowest strength values were achieved with loading along the z-direction.

### 3.3. Shear Tests

**Figure 11:**
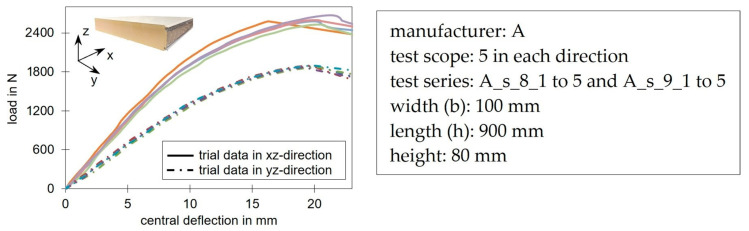
Deflection-load curves of shear beams along (*x*-axis) and transverse to the production direction (*y*-axis). The different colors represent the individual trials of the test series.

Figure 11 shows the shear test setup and the associated typical load-deflection curves for shear beams along and transverse to the production direction. Both test directions had a linear-elastic material behavior at the beginning before a non-linear behavior appeared. A direct comparison of the load levels was not possible here, as the specimens were from panels with slightly profiled face sheets, which caused a slight stiffening effect for the beams along the x-axis. Nevertheless, the comparison of tests loaded in different spatial directions showed that the overall deformation was almost identical, while the ultimate loads and the stiffnesses of the xz-plane were significantly higher. Results from Grimm [39] on square joint tests, in which the face sheets have no influence, show a reduction in strength from 29% to 34% and a reduction of the shear modulus from 36% to 43% in the transverse direction.

The failure in this test setup is usually a shear failure in the core material, which occurs suddenly (Figure 4a). The failure load and the dimensions were used to calculate the maximum shear stress that could be applied. The shear modulus was determined in the linear-elastic range. In those shear tests, generally, deformations occurred from the bending of the face sheets as well as from the shear deformation of the core. The shear modulus was only determined from the deformation component of the shear-stressed core in the linear deformation range.

Occasionally, delamination of the face sheets and indentations in the core also occurred as a form of failure. The bearing loads resulting from these failures were neglected to determine the shear characteristics.

Similar to the compression and tensile tests, the linear load-deformation in the beginning resulted from the bending of the cell bars (analogous to Figure 8b). This was followed by a buckling of the cell bars (Figure 8c), as compressive and tensile stresses were superimposed in the shear tests. However, in contrast to the compression tests, the shear tests did not have a densification area. The main stress component, which ran approximately between the support and the load action, resulted in tensile failure or shearing of the cell webs. The higher initial stiffness in the xz-plane compared to the yz-plane matched the increased compressive and tensile stiffness in the x-direction. The higher load-bearing capacity in this plane also matched the higher strength values from the compression and tensile tests.

The deviation of the load-deformation curve from the linear curve also appeared in the investigations of shear tests of other sandwich panels. Some of these had different dimensions as well as face and core materials. In some cases, the deviation of the linear deformation curve is attributed to the yielding of the face sheets [28,29]. This was not the case in the tests presented here. Analytical calculations and numerical modeling by the authors showed that the steel faces were always in their linear-elastic range and therefore did not lead to the curvature in the load-deformation diagram.

### 3.4. Bending Capacity Tests

**Figure 12:**
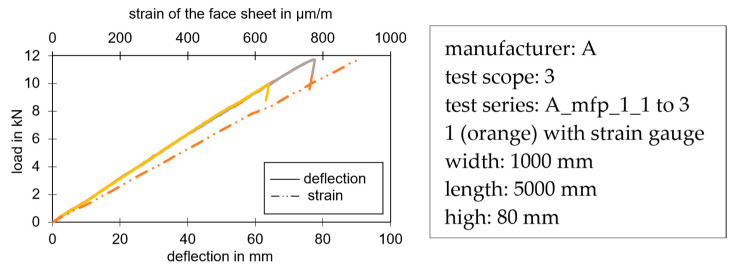
Typical load-deflection curves. The different colors represent the individual trials of the test series. One sample (orange) with strain measurement in the middle on the bottom steel face sheet.

Compared to the diagrams of the compression and shear tests, the bending capacity tests showed an almost linear behavior until failure. The strain measurement using a strain gauge in the middle of the panel on the bottom face sheet also showed an almost linear curve (see Figure 12).

The failure that occurred in the visualized test series was a wrinkling of the compressed face sheet. The maximum bending capacity up to this failure could be determined experimentally via the described test or analytically via the material parameters from the small part tests. Shear failure in the core could also occur in the tests between the support and the first loading point which then determined the maximum load-bearing capacity. This failure mode occurred more and more frequently with the current foam systems, particularly after temperature loading. Thus, with structural changes in the core, the shear failure becomes the decisive failure mode [31].

## 4. Numerical Investigation

The numerical analysis presented in this paper on hand was carried out with Ansys 2022 R2 Workbench [40]. The test setups were implemented to validate the selected material parameters. The geometric and material non-linearity was taken into account. The material-dependent non-linearity was particularly evident in the compression and shear tests from the experimental investigations (Figure 6 and Figure 11). The geometric non-linearity was necessary due to the sometimes large deformations in comparison to the sample dimensions.

### 4.1. Implementation

The steel face sheets were neglected in the analysis here. Since only compression and tensile tests were analyzed in the numerical study, the steel faces had no influence on the results.

Particular attention was paid to the modeling of the closed-cell PU rigid foam core. Volume elements were used for this purpose. In the experimental investigations presented earlier in this paper, various characteristics (stiffness, strength, orthotropy) were identified that could be implemented in a detailed numerical or analytical investigation. A model that included manufacturer-independent material data was not possible due to the high influence of the production process and the different mixing ratios of the raw materials (polyol, isocyanate, and additives) on the properties. Nevertheless, some general relationships could be established between load and deformation.

Due to the aim of mapping the entire load-bearing behavior up to fracture, the complete material range of the PIR must also be presented as far as possible. This includes, in particular, the non-linear range appearing in the compression tests.

Figure 5 and Figure 9 show that the material behavior between tension and compression was different. The core material behaved almost hyper-elastically under compression, whereas under tension it behaved linearly and failed due to being brittle. Nevertheless, the load directions were not defined separately in the presented model. Due to the lower tensile strength compared to the compressive strength at yield and the almost identical stiffnesses in the linear tensile and compressive range, the tensile behavior could be defined similarly to the initial range under compression. Furthermore, the PIR foam did not behave isotropically. The relationship between the modulus of elasticity and the shear modulus via Poisson’s ratio ($E_{C}=2\text{⋅}(1+v)\text{⋅}G_{C}$) was not applicable for PU [5,41].

Based on various preliminary investigations, and in order to obtain a numerical model that is as stable as possible, the PU rigid foam was initially defined with two different parts: the linear elastic range using orthotropic elasticity and the non-linear part, which appeared in the compression tests and was implemented using the Hill yield criterion.

For this purpose, the engineering stresses and strains resulting from the tests were first converted into true stresses and strains for implementation in Ansys. This was performed under the assumption of volume constancy. For the data obtained from the compression tests, the following correlations between the engineering data (σ, ε) and the true values ($\sigma_{w}$, $\varepsilon_{w}$) can be seen [42]:

(1) $$\begin{array}{cc} \sigma_{w}=\sigma \text{⋅}(1-\varepsilon ) & \varepsilon_{w}=\ln\left(1+\varepsilon \right) \end{array}$$

**Figure 13:**
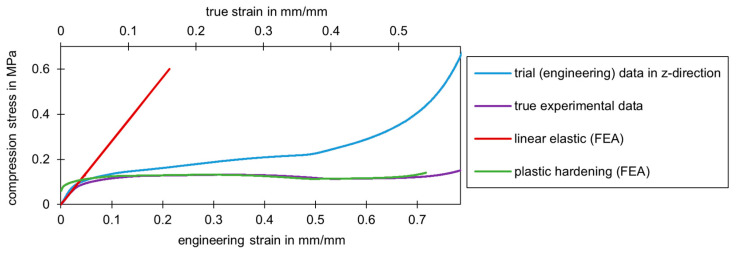
Experimental engineering and true data from a compression test (manufacturer A) and the resulting different two areas (linear elastic and plastic hardening) for implementation in a numerical model.

Figure 13 shows the stress-strain curve for a compression test in the z-direction. The curve of the technical data (blue curve) obtained directly from the test and the true stress-strain curve (purple curve), calculated according to Equation (1), are shown. In addition, the linear elastic range (red line) and the plastic hardening (green curve) implemented in the numerical model are visualized. The assumption of the calculation of the volume constancy used to convert the engineering data into the true data was sufficiently accurate as long as the two graphs did not run contrary to each other. Figure 11 shows that this point in time occurred at a technical strain of around 25%. From this point onwards, the true strains decreased while the technical strains continued to increase. The assumption of volume constancy was no longer valid. It can be assumed that a large proportion of the cell walls had already burst at this strain. Consequently, the material was no longer predominantly a closed cell and the densification began. Results from the modeling were only used up to a maximum strain of around 25%. This material model is not suitable for data beyond this range.

If the representation of a high strain is desired, a hyper-elastic material model can be used, for example. In this case, the test data for the material model was also implemented. The different material behavior of the material parameters in the various loads was more difficult to represent here. Convergence in the calculation was also sometimes worse with hyper-elastic models. For this reason, the material model in this paper was based on the linear-elastic initial range with a subsequent plastic hardening range.

The plastic hardening area was implemented in Ansys with the help of the Hill yield criterion. The Hill yield criterion is a way of determining yield stress levels in different spatial directions and planes. It was developed by Rodney Hill in 1950 and is a suitable criterion for describing anisotropic plastic deformations. Six constants have to be defined for this purpose [43]:

(2) $$\begin{array}{ccc} R_{\mathrm{xx}}=\frac{\sigma_{x}^{Y}}{\sigma_{0}} & R_{\mathrm{yy}}=\frac{\sigma_{y}^{Y}}{\sigma_{0}} & R_{\mathrm{zz}}=\frac{\sigma_{z}^{Y}}{\sigma_{0}}\\ R_{\mathrm{xy}}=\frac{\tau_{\mathrm{xy}}^{Y}}{\tau_{0}}=\sqrt{3}\text{⋅}\frac{\tau_{\mathrm{xy}}^{Y}}{\sigma_{0}} & R_{\mathrm{xz}}=\frac{\tau_{\mathrm{xz}}^{Y}}{\tau_{0}}=\sqrt{3}\text{⋅}\frac{\tau_{\mathrm{xz}}^{Y}}{\sigma_{0}} & R_{\mathrm{yz}}=\frac{\tau_{\mathrm{yz}}^{Y}}{\tau_{0}}=\sqrt{3}\text{⋅}\frac{\tau_{\mathrm{yz}}^{Y}}{\sigma_{0}} \end{array}$$

The constants shown in (2) must be determined using the experimental data. The relationship between the normal and shear stress is defined by $\tau_{0}=\frac{1}{\sqrt{3}}\text{⋅}\sigma_{0}$.

This model does not differentiate between the load direction tension and compression. In addition, the hardening in the form of stress-strain relationships cannot be defined separately for the individual directions and planes. Only the point of yielding can be adjusted for every direction via Equation (2) using the experimental data.

### 4.2. Validation

With the help of the Hill yield criterion, it was possible to reconstruct the trial data of the compression and tensile test. The parameters were calculated with the help of the yield points from the experimental data of the three spatial directions. The stress-strain relationship in the non-linear area was calibrated at the compression test in z-direction which was also the dominant loading direction for the core of sandwich panels as roof and façade components.

**Figure 14:**
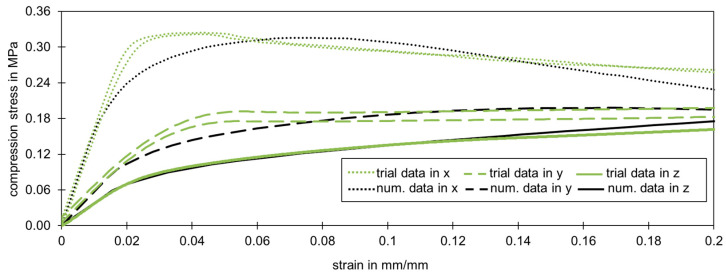
Comparison of the compression trial data and the numerical data (using the Hill yield criterion calibrated on the compression test in z-direction) (manufacturer A).

Figure 14 shows the correlation, particularly in the initial range up to a compression strain of 20%. Regarding the dominant loading direction, the best fit was in the z-direction up to 10% strain. At higher strains, the numerical model could be used due to the validity of the previously defined and used assumption of volume constancy in the calculation of the true stresses and strains. Since this range is sufficiently large to adequately represent the main deformation, this model will be used in further investigations.

**Figure 15:**
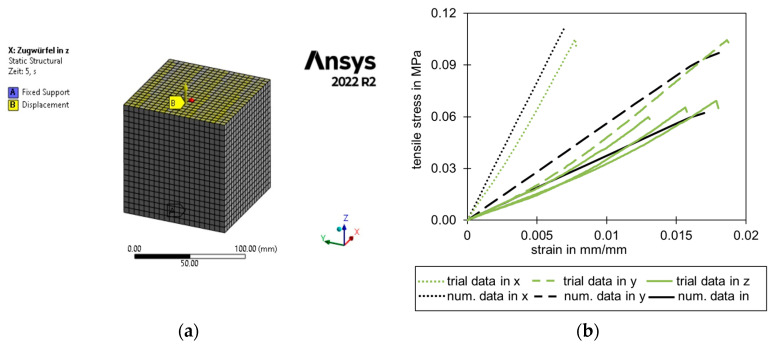
(**a**) Numerical model of a cube test specimen; (**b**) Comparison of the tensile trial data and the numerical data (using the Hill yield criterion calibrated on the compression test in z-direction) (manufacturer A).

Figure 15a shows the FEA model of a cube tensile test. The tensile data fit in an acceptable range using the described material model, which was calibrated on the compression test. Taking the large scatter into account, which can be observed in the trial data in the z-direction in Figure 15b, the numerical model was within an acceptable range for sandwich panels used in construction. The failure stresses and mechanisms can be determined using the approaches discussed below.

## 5. Discussion

The experimental investigations show a wide range of results that have not yet been investigated in detail in this form for current sandwich panels in the construction industry. The various types of failure and the resulting maximum loads are of great interest to the construction industry. The experimental investigations showed different failure modes. At the cellular level, the cell bars tore, and the resulting tensile fracture occurred. In the compression tests, the collision of the buckling cell walls at high compression strain could be defined as failure (densification). Well before the cell bars failed, the cell walls usually tore open in the area of high strains. This allowed the blowing agent to escape, particularly in the case of test specimens with large exposed core faces compared to their volume. This behavior of the cells combined with the overall load-bearing behavior of sandwich panels led to different failure patterns in the overall system.

Of particular relevance is the wrinkling of the steel faces and the core fractures. Wrinkling occurred mainly in the bending capacity tests when the specimen was primarily subjected to bending. This bending generated normal stresses in the face sheet, which led to a stability failure of the face that was under compression and supported by the core. For experimental determination, the bending tests described in Section 2.2.4 were carried out. Analytically, the maximum wrinkling stress was determined via the material parameters of the core (E_C_, G_C_), which resulted from the small-scale tests, and the stiffness of the face sheets (E_F_) [5].

(3) $$\sigma_{w}=k\text{⋅}\sqrt[{3}]{E_{F}\text{⋅}E_{C}\text{⋅}G_{C}}$$

The constant k is usually set at 0.5 or 0.82 [5]. The requirement for this failure case is that the shear stress in the core is still below the bearable shear strength. The stiffness values of the core and the face sheets in the linear-elastic range were used as the basis for calculating the wrinkling stress. This assumption is generally correct for the face sheets made of steel. The small-part tests showed that the core material leaves the linear range under compression at a strain of 3% (see Figure 5). Therefore, the calculation for the wrinkling stress was only reliable as long as the core material was in the elastic range. Otherwise, Equation (3) has to be modified by using the significantly lower stiffnesses of the plastic range. If the resistance to wrinkling is so high that plasticization of the core would initially occur below the compressed face sheet, the core material often fails first in the shear region [26]. The wrinkle failure can be recognized in the numerical model by sudden strong deformations that cause the model to no longer converge. Investigations show that the failure criterion of wrinkling, which dominated in the past, is no longer the sole decisive mode of failure. Shear failure of the core has occurred more frequently in the recent past [31]. This fits in with the damage patterns that the sandwich industry is currently struggling with. It often occurs after installations and loading. This is a failure of the core layer that occurs below the resistance values described in the approvals.

The core failure of the PIR rigid foam occurs when the maximum stress in tension, shear, compression, or multiaxial constellation exceeds the resistance. This failure can be determined in the numerical calculations via the stress tensors of the core and their state with the use of the Tsai–Wu failure criterion [26]. Compared to the Tsai–Wu failure criterion, the often classically used von Mises failure criterion is limited to primarily isotropic materials and is only a rough approximation for highly anisotropic materials. The shear stresses are taken into account in the von Mises criterion via the equivalent stresses. In contrast, the shear stresses in the Tsai–Wu criterion are considered in a more complex way via the specific material parameters for the shear stress. However, the more specific Tsai–Wu failure criterion can be converted into the von Mises failure criterion in certain cases.

Tsai and Wu published a strength criterion for anisotropic materials in 1971 [44]. The Tsai–Wu failure criterion can be used to describe the stress failure in the core. This failure criterion is characterized by the consideration of structural anisotropy and a distinction between compressive and tensile strengths. For example, the criterion is used to determine the failure of fiber composites. However, it can also be applied to foam materials. With the simplified Tsai–Wu criterion, the parameters can be reduced, and the failure surface can be represented for a plane (e.g., xz or xy) as an ellipsoid in the 2D plane. The Tsai–Wu criterion can be expressed in relation to the xz plane as follows [45]:

(4) $$f_{x}\cdot \sigma_{x}+f_{z}\cdot \sigma_{z}+f_{\mathrm{xx}}\cdot {\sigma_{x}}^{2}+f_{\mathrm{zz}}\cdot {\sigma_{z}}^{2}+2{\text{⋅}f}_{\mathrm{xz}}\cdot \sigma_{x}\cdot \sigma_{z}=1-k^{2}$$

Using the parameters:

(5) $$\begin{array}{ccc} f_{x}=\frac{1}{F_{\mathrm{xt}}}-\frac{1}{F_{\mathrm{xc}}} & f_{z}=\frac{1}{F_{\mathrm{zt}}}-\frac{1}{F_{\mathrm{zc}}} & f_{\mathrm{xx}}=\frac{1}{F_{\mathrm{xt}}\cdot F_{\mathrm{xc}}}\\ f_{\mathrm{zz}}=\frac{1}{F_{\mathrm{zt}}\cdot F_{\mathrm{zc}}} & f_{\mathrm{xz}}=-\frac{1}{2}{\left(f_{\mathrm{xx}}\cdot f_{\mathrm{zz}}\right)}^{\frac{1}{2}} & \tau_{\mathrm{xz}}=k\text{⋅}F_{\mathrm{xz}} \end{array}$$

$F_{\mathrm{xt}}$ and $F_{\mathrm{xc}}$ describe the tensile and compressive strengths in the x-direction. Similarly, $F_{\mathrm{zt}}$ and $F_{\mathrm{zc}}$ result from the strengths in the z-direction. $F_{\mathrm{xz}}$ indicates the shear strength. These parameters can be obtained from pure compression, tensile, and shear tests. $\sigma_{x}$, $\sigma_{z}$ and $\tau_{\mathrm{xz}}$ represent the normal and shear stresses in the respective direction or plane. The failure curve can be represented by ellipses in the 2D space with different constant shear stress levels represented by the constant k.

**Figure 16:**
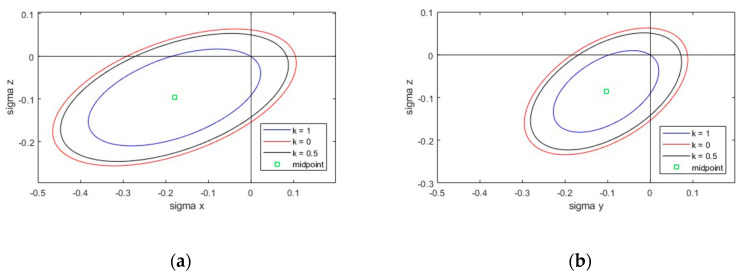
Representation of the yield surface by using the Tsai–Wu failure curve for a PIR foam system (manufacturer A) with different shear stress levels (k = 0, k = 0.5, and k = 1): (**a**) in the xz-plane; (**b**) in the yz-plane.

Figure 16 shows the ellipsoidal failure curve for the xz- and yz-plane according to the Tsai–Wu criterion for a PIR foam system with different shear stress levels. It can be seen that the ellipses extended far into the compression-compression quadrant. According to Figure 16, the most critical area was the tension-tension quadrant. The most critical load combination was the tension-shear.

In the xz-plane, the center of all ellipses resulted in k = 1.42. Consequently, the material could absorb shear stresses in this plane that were up to 42% higher than the pure shear strength. Looking at the shear test in Figure 11, the load-deformation curve deviated from the linear initial range at around 60% of the ultimate load. This initial yield fits well with the k-value of 1.42. It can be assumed that the shear test described in EN 14509 does not only trigger pure shear stresses in the core material. Local transverse compression of the core material, due to load and support areas, results in greater absorbable shear stresses than the pure shear strength of the material. In the yz-plane, a biaxial stress state resulted in up to 19% higher stresses than the pure shear stresses that could be absorbed in this plane. It is clear that the classical stress theory according to von Mises, which is often used as a basis, cannot be applied to PIR rigid foam.

The bending capacity tests described above can also fail due to a shear core fracture. In this case the recalculation results in significantly lower absorbable shear stresses than in the shear beam tests. This may also indicate a higher biaxial stress state of the shear beams. Due to the compressed areas in their test setup, the maximum shear stress is increased compared to the pure shear stress.

Based on the sandwich load-bearing effect in which the core material receives pure constant shear stresses, and the normal stresses are transferred via the face sheets, the failure appears to be caused by a core fracture when the maximum shear strength is reached. However, due to the concentrated load introduction and dimensions of the specimens, normal stresses also occur in the core material. The core is then biaxially loaded, and the failure can be described in this area using the Tsai–Wu criterion with a suitable k-value. Hassinen 1999 [46] already mentioned investigations of two-span sandwich panels in which it was found that the von Mises flow criterion does not apply to PU rigid foams. Hassinen showed that PU actually has higher capacities with the simultaneous effect of shear and compression than the von Mises criterion estimates.

The criterion can only describe the core failure of the foam system. Failure maps usually take into account the geometric parameters of the test specimens in order to differentiate core failure (via Tsai–Wu) from wrinkling failure [26].

Further investigations and more detailed studies of the material behavior and the stress states of sandwich panels during loading are necessary. Even today, tests are planned to record the stress and distortion distributions in specimens with the aid of digital image correlation (DIC) measurements. With these measurements, the values for the yield stress correlations described in Equation (2) can be calibrated more precisely and the proposed Tsai–Wu failure criterion can be validated. Furthermore, the inhomogeneities and their influence on the stress distribution can be recorded with the aid of DIC measurements herewith. It should be possible to validate the numerical model and the implemented failure mechanisms in more detail. The investigation of a hyper-elastic material model or other suitable models can also be considered.

## 6. Conclusions

This article describes the material behavior of sandwich panels, consisting of steel faces and a PU rigid foam (40 kg/m^3^) core, in the construction industry under different loads, as a result of a large number of tests and beyond the standard. Particular attention was paid to the core material PIR rigid foam. The most important results are listed below:

- The experimental investigations illustrated the strong directional dependence of the foam system as well as its different behaviors depending on the type of loading (compression, tension, or shear). The orthotropy was caused in particular by the manufacturing process. The process led to different cell geometries along and transverse to the production. This led to various properties along the three spatial directions. The tests specified in the standard were not sufficient for a detailed description of the core material. The results showed that tests in all spatial directions are essential for understanding the behavior of the material.
- In compression tests an almost hyper-elastic material could be found in y- and z-direction. The foam could be compressed in these directions by up to 90% without any cracks. In the x-direction, the core material behaved more like an elastic-plastic foam material. Cracks in the foam occurred even with small compression. The maximum strength and stiffness were detected in the production direction.
- Under tensile stress, PIR failed as a brittle material with low tensile strength. The maximum strength and stiffness appeared in the production direction. The tensile strength was generally lower than the compression strength.
- In addition to the linear range in the stress-strain curve, the shear tests also showed a non-linear range. This was caused by the core material and matched the results from the compression tests. It can be assumed that the shear test described in EN 14509 does not only trigger pure shear stresses in the core material.
- In the numerical model presented in this paper, an orthotropic elasticity with plastic hardening according to the Hill yield criterion was used to represent the PIR rigid foam. The orthotropic plastic range of the compression tests could be well represented by adjusting the yield stress levels of the different directions and planes.
- The tests suggest that the classical failure criterion according to von Mises is not valid for the PIR foam used in the investigated sandwich panels. The Tsai–Wu criterion is proposed here, which considers the orthotropy of the material as well as the different tensile and compression strengths. This criterion can be used to predict the failure in the core due to a biaxial state with shear.

## Figures and Tables

**Table 1:** Properties of the tested sandwich panels.

	Manufacturer A	Manufacturer R
**Type**	wall panel	roof panel
**Outer face sheet**		
material	steel	steel
thickness ^1^	0.55 mm	0.53 mm
profiling ^1^	micro (depth 0.79 mm)	profiled (depth 43.03 mm)
**Outer face sheet**		
material	steel	steel
thickness ^1^	0.45 mm	0.46 mm
profiling ^1^	line (depth 0.87 mm)	line (depth 1.23 mm)
**Core**		
material	PIR rigid foam	PIR rigid foam
thickness ^1^	80 mm and 160 mm	100 mm and 170 mm
density ^1^	40.20 kg/m^3^ (80 mm) 41.02 kg/m^3^ (160 mm)	39.02 kg/m^3^ (100 mm) 40.76 kg/m^3^ (170 mm)

^1^ Mean value out of a minimum of 6 measurements.

## Data Availability

The data presented in this study are available on request from the corresponding author.
